# Triglyceride-glucose index in early pregnancy predicts the risk of gestational diabetes: a prospective cohort study

**DOI:** 10.1186/s12944-024-02076-2

**Published:** 2024-03-25

**Authors:** Yufeng Guo, Junwen Lu, Mailiman Bahani, Guifeng Ding, Lei Wang, Yuxia Zhang, Huanmei Zhang, Chengyao Liu, Lijun Zhou, Xiaolan Liu, Fangshen Li, Xiaoli Wang, Hong Ding

**Affiliations:** 1 Department of Public Health, Xinjiang Medical University, Urumqi, Xinjiang Uygur Autonomous Region 830000 China; 2 Urumqi Maternal and Child Health Hospital, Urumqi, Xinjiang Uygur Autonomous Region 830000 China; 3 Department of Maternal and Child Nutrition, National Institute for Nutrition and Health, Chinese Center for Disease Control and Prevention, Beijing, 100050 China; 4 Maternal and Child Health Care Hospital of Xinjiang Uygur Autonomous Region, Urumqi, Xinjiang Uygur Autonomous Region 830000 China

**Keywords:** Early pregnancy, Triglyceride-glucose index, Gestational diabetes mellitus, Pregnancy-related complications, Singleton pregnancy

## Abstract

**Objective:**

This study aimed to investigate the association between the triglyceride-glucose (TyG) index in early pregnancy and the development of gestational diabetes mellitus (GDM) in the second trimester. The primary objectives were to evaluate the predictive potential of the TyG index for GDM, determine the optimal threshold value of the TyG index for GDM assessment, and compare the predictive performance of the TyG index alone versus its combination with maternal age and pre-pregnancy body mass index on GDM. Moreover, the study explored the association between the TyG index in early pregnancy and the risk of other pregnancy-related complications (PRCs), such as placental abruption and gestational hypertension.

**Patients and methods:**

This prospective cohort study recruited 1,624 pregnant women who underwent early pregnancy antenatal counseling and comprehensive assessments with continuous monitoring until delivery. To calculate the TyG index, health indicators, including maternal triglycerides and fasting plasma glucose, were measured in early pregnancy (< 14 weeks of gestation). The predictive power of the TyG index for evaluating GDM in Chinese pregnant women was determined using multifactorial logistic regression to derive the odds ratios and 95% confidence interval (CI). Subgroup analyses were conducted, and the efficacy of the TyG index in predicting PRCs was assessed via receiver operating characteristic (ROC) curve analysis and restricted cubic spline, with the optimal cutoff value calculated.

**Results:**

Logistic regression analyses revealed a 2.10-fold increase in the GDM risk for every 1-unit increase in the TyG index, after adjusting for covariates. The highest GDM risk was observed in the group with the highest TyG index compared with the lowest quintile group (odds ratios: 3.25; 95% CI: 2.23–4.75). Subgroup analyses indicated that exceeding the recommended range of gestational weight gain and an increased GDM risk were significantly associated (*P* = 0.001). Regarding predictive performance, the TyG index exhibited the highest area under the curve (AUC) value in the ROC curve for GDM (AUC: 0.641, 95% CI: 0.61–0.671). The optimal cutoff value was 8.890, with both sensitivity and specificity of 0.617.The combination of the TyG index, maternal age, and pre-pregnancy body mass index proved to be a superior predictor of GDM than the TyG index alone (AUC: 0.672 vs. 0.641, *P* < 0.01). After adjusting for multiple factors, the analyses indicated that the TyG index was associated with an increased risk of gestational hypertension. However, no significant association was noted between the TyG index and the risk of preeclampsia, placental abruption, intrauterine distress, or premature rupture of membranes.

**Conclusion:**

The TyG index can effectively identify the occurrence of GDM in the second trimester, aligning with previous research. Incorporating the TyG index into routine clinical assessments of maternal health holds significant practical implications. Early identification of high-risk groups enables healthcare providers to implement timely interventions, such as increased monitoring frequency for high-risk pregnant women and personalized nutritional counseling and health education. These measures can help prevent or alleviate potential maternal and infant complications, thereby enhancing the overall health outcomes for both mothers and babies.

**Supplementary Information:**

The online version contains supplementary material available at 10.1186/s12944-024-02076-2.

## Background

Gestational diabetes mellitus (GDM) arises from disruptions in glucose metabolism, resulting in elevated blood glucose levels during pregnancy. This condition poses significant risks for both mothers and children, including pregnancy complications, birth anomalies, and long-term health issues [1–3]. A meta-analysis involving 79,064 Chinese participants across 25 studies revealed a GDM prevalence of 14.8% in China [4]. Early identification and effective management of GDM are essential to safeguard the health of both mother and child. Currently, the primary diagnostic method for GDM is the oral glucose tolerance test performed between 24 and 28 weeks of gestation. However, the fetus may be exposed to intrauterine hyperglycemia before 24 weeks, highlighting the importance of early diagnosis. Delayed diagnosis between 24 and 28 weeks cannot fully reverse the adverse effects of intrauterine hyperglycemia on offspring. Additionally, most GDM patients do not exhibit obvious symptoms before diagnosis [5, 6], particularly in early pregnancy (before 14 weeks), making early detection challenging. Therefore, effective predictive methods for GDM in early pregnancy are crucial.

Insulin resistance (IR) is a critical factor in the development of GDM, heightening the susceptibility of expectant mothers and their offspring to metabolic disorders, chronic inflammation, and related conditions such as diabetes mellitus, hypertension, and cardiovascular disease [7–10]. The triglyceride-glucose (TyG) index, calculated as follows: Ln [fasting plasma glucose (FPG) (mg/dL) × fasting triglyceride (mg/dL)/2] [11], has emerged as a reliable surrogate marker for identifying IR and metabolic disorders due to its simplicity and practicality [12–14]. It is widely utilized in clinical settings to assess GDM risk. Various TyG indices have been established to identify women vulnerable to GDM in different regions, including southeastern and northern China [15], Mexico [16], Korea [17], Hungary [18], and Iran [19]. However, current studies on pregnant women have several methodological shortcomings, such as reliance on a single sample source, small sample sizes and subgroups, insufficient control of confounding variables, and the absence of universally accepted cutoff values for the TyG index in expectant mothers.

This study represents the first application of the TyG index to predict the incidence of GDM in northwest China (Urumqi). Its objective is to determine the optimal cutoff value of the TyG index for predicting GDM risk among pregnant women in early pregnancy while controlling for confounding variables. In contrast to previous research, it seeks to offer fresh insights into the predictive performance difference between the independent TyG index and the combination of the TyG index with age and pre-pregnancy body mass index (BMI). Utilizing logistic regression methods, the study aims to explore the feasibility of assessing GDM risk using the TyG index in early pregnancy after adjusting for potential covariates such as maternal ethnicity, pre-pregnancy BMI, age, assisted reproduction, miscarriage history, pregnancy, parity, gestational age, and gestational weight gain (GWG). Subgroup analyses were conducted based on maternal age, number of pregnancies, miscarriage history, assisted reproduction, pre-pregnancy BMI, GWG, and preterm birth. The difference in predictive performance between the independent TyG index and the combination of the TyG index with age and pre-pregnancy BMI was evaluated using the area under the receiver operating characteristic (ROC) (AUC) curve. If proven reliable, this approach can establish a comprehensive outpatient monitoring system for pregnant women with high TyG index levels in early pregnancy, facilitating timely interventions to improve pregnancy outcomes.

## Materials and methods

### Data source

Data from the Science and Technology Basic Resources Survey Special Project-China Maternal Nutrition and Health Scientific Survey (Northwest China Project Site) was used. This population-based prospective cohort study was conducted among pregnant women residing in Urumqi, northwest China, for an extended period. The cohort was established between August 2021 and April 2023, with pregnant women having an average age of (31.7 ± 4.0) years. Primiparous women constituted 64.1% of the sample.

The inclusion criteria for this cohort study encompassed pregnant women aged 18 years or older, with a gestational age of less than 14 weeks, engaging in prenatal care for the first time, having signed an informed consent form, and demonstrating the ability to accurately understand and independently respond to the researchers’ questions. The exclusion criteria comprised women diagnosed with severe cardiovascular and cerebrovascular diseases, liver and kidney diseases, mental disorders, intellectual disabilities, or those unable to meet the study’s requirements independently. Additionally, women with pre-GDM were excluded based on medical history inquiry and blood glucose testing, following the guidelines of the International Association of the Diabetes and Pregnancy Study Groups. Similarly, pregnant women with hypertension before pregnancy or presenting with a systolic blood pressure (SBP) of ≥ 140 mmHg and/or a diastolic blood pressure (DBP) of ≥ 90 mmHg before 20 weeks of gestation, diagnosed as hypertension complicating pregnancy according to the definition provided by the International Society for the Study of Hypertension in Pregnancy, were also excluded from participation in the study. The final study population comprised 1,624 women with singleton pregnancies (Supplementary Fig. 1).

This study received approval from the Ethics Committee of the Institute of Nutrition and Health of the Chinese Centre for Disease Control and Prevention (Approval No. 2021-008) and adhered to the guidelines of the Declaration of Helsinki. The participants were adequately informed about the study’s objectives and procedures and provided written consent.

### Measurements and definitions

#### Data collection

During the first trimester of pregnancy (gestational age < 14 weeks), basic information questionnaires and biomarker measurements were collected. For women with regular menstrual cycles, fetal age was calculated from the first day of the last menstrual period to the current time. For those with irregular cycles, early pregnancy ultrasound examinations were conducted to estimate gestational age based on the size of the gestational sac and length of the embryonic bud. Based on this estimation, the last menstrual period can be inferred, and the fetal age can be calculated. Blood pressure was measured after a minimum 5-min rest in a sitting or lying position, using an upper arm blood pressure monitor to measure the right brachial artery blood pressure. Three measurements were taken, and the average was recorded. Blood samples were collected after a minimum 8-h fast for analysis of biomarkers including FPG (mg/dL), triglycerides (mg/dL), total cholesterol (TC, mg/dL), low-density lipoprotein cholesterol (LDL-C, mg/dL), high-density lipoprotein cholesterol (HDL-C, mg/dL), hemoglobin (g/L), uric acid (UA, μmoI/L), urine creatinine (μmoI/L), alanine aminotransferase (ALT, U/L), and aspartate aminotransferase (AST, U/L). The TyG index was also calculated. After delivery, data on pregnancy examinations and outcomes were collected.

#### Oral glucose tolerance test

The oral glucose tolerance test was conducted following the Chinese GDM diagnosis guidelines (2014). Pregnant subjects in their 24th–28th week of gestation maintained a normal diet for 3 days prior to their hospital visit, consuming no less than 150 g of carbohydrates daily and fasted for at least 8 h before the oral glucose tolerance test. During the examination, they remained seated and refrained from smoking. Subjects ingested 300 mL of a water solution containing 75 g of glucose within 5 min. Venous blood samples were collected before ingestion and at 1 and 2 h after ingestion (timing starts from the initial ingestion of the glucose solution) for glucose level measurement using the glucose oxidase method. A diagnosis of GDM was made if any of the following criteria were met or exceeded: a fasting blood glucose level of 5.1 mmol/L (91.90 mg/dL), a 1-h blood glucose level of 10.0 mmol/L (180.20 mg/dL), or a 2-h blood glucose level of 8.5 mmol/L (153.17 mg/dL). These criteria are consistent with the latest guidelines from the International Association of the Diabetes and Pregnancy Study Groups [20]. The definitions of gestational hypertension (GH) and preeclampsia are referred to the International Society for the Study of Hypertension in Pregnancy. The definitions of pregnancy-related complications (PRCs) other than GDM and hypertensive disorders were referred to International Classification of Diseases-10.

#### Other covariates

In addition to the primary variables, the study considered several other covariates known to impact outcome measures, including ethnicity, maternal age, pre-pregnancy BMI, assisted reproduction, abortion history, gravidity, parity, GWG, and gestational week of examination. Ethnicity and age were obtained from participants’ identification cards. Pre-pregnancy BMI was calculated by dividing pre-pregnancy weight (kg) by height squared (m). Fertility status was determined using information from participants’ questionnaires and obstetric/gynecological medical records.

According to the 2009 guidelines from the Institute of Medicine in the United States [21], recommended GWG is categorized as follows: for underweight individuals (BMI < 18.5 kg/m^2^), a weight gain of 12.5–18 kg during pregnancy; for normal weight individuals (18.5 ≤ BMI < 25 kg/m^2^), a weight gain of 11.5–16 kg during pregnancy; for overweight individuals (25 ≤ BMI < 30 kg/m^2^), a weight gain of 7–11.5 kg during pregnancy; and for obese individuals (BMI ≥ 30 kg/m^2^), a weight gain of 5–9 kg during pregnancy. Based on these guidelines, weight gain during pregnancy can be classified as: i) below the recommended range; ii) within the recommended range; or iii) above the recommended range.

### Statistical analysis

The normal distribution of continuous variables was assessed using the Kolmogorov–Smirnov test. Continuous variables with a normal distribution were presented as mean ± standard deviation, while those not following a normal distribution were expressed as median (25th percentile, 75th percentile). Categorical variables were presented as frequencies and percentages. Differences in continuous variables between groups were compared using a one-way analysis of variance or the Kruskal–Wallis rank-sum test, while the chi-square test was employed to compare differences in categorical variables between groups.

To investigate the association between the TyG index and GDM, a multifactorial logistic regression analysis was conducted. The TyG index was evaluated both per unit and by quintile, with the lowest quintile serving as the reference. Two adjustments were made for potential confounding factors. Model 1 adjusted for ethnicity, pre-pregnancy BMI, maternal age, assisted reproduction, miscarriage history, gravidity, parity, gestational age at delivery, and GWG. Model 2 further adjusted for gestational age at examination, SBP, DBP, TC, LDL-c, HDL-c, hemoglobin, UA, urine creatinine, ALT, and AST. Subsequently, a linear trend assessment was performed on the model after evaluating the TyG index by quintiles.

To expand the understanding of the association between the TyG index and GDM risk, subgroup analyses were conducted based on maternal age (< 30, 30–34, or ≥ 35 years), number of pregnancies (≤ 2, > 2), miscarriage history (no, yes), assisted reproduction (no, yes), pre-pregnancy BMI (underweight, normal weight, overweight, and obese), GWG (within or above recommended range), and preterm birth (no, yes). Interactions across these subgroups were evaluated using likelihood ratio tests to compare whether there were differences in effects among the subgroups.

Moreover, the possible nonlinear correlation between TyG index changes and PRCs was examined through restricted cubic spline analysis using the rms package in R software. Furthermore, to evaluate the predictive performance of the TyG index for PRCs, ROC curves were plotted, and AUC values were calculated using the pROC package in R software, with 95% confidence intervals (CI) computed using the bootstrap method. This study also compared the predictive ability of the “TyG index alone” and the “TyG index combined with maternal age and pre-pregnancy BMI” for GDM.

All statistical procedures were performed using SPSS 25.0, R 4.2.2, and MSTATA (https://www.mstata.com/). SPSS was utilized for descriptive statistics, regression analysis, and other statistical procedures, while R and MSTATA were employed for data visualization. A* P*-value of < 0.05 was considered statistically significant, while a *P*-value of < 0.01 was deemed highly statistically significant.

## Results

### Baseline characteristics

The study included 1,624 pregnant women with an average age of 31.7 ± 4.0 years. The majority were of Han Chinese ethnicity (62.4%), and 48.4% were experiencing their first pregnancy. Among them, 447 developed GDM, resulting in a GDM prevalence of 27.52%. As the TyG index increased, significant increases in maternal age, gravidity, parity, and DBP were observed (*P* < 0.05). Similarly, pre-pregnancy BMI, assisted reproduction, abortion history, SBP, FPG, triglycerides, TC, hemoglobin, UA, ALT, and AST also significantly increased with the TyG index (*P* < 0.01). Conversely, the duration of gestation during delivery decreased with an increasing TyG index (*P* < 0.05). Gestational week at examination, LDL-C, HDL-C, and GWG showed a significant trend of first increasing and then decreasing with the TyG index (*P* < 0.01). No significant differences were observed in ethnicity and urine creatinine across quintiles of the TyG index (Table 1).

**Table 1:** Anthropometric and biochemical characteristics of the participants at baseline according to the TyG index quintiles

Variables	Overall	TyG Index Quintiles					*P* value
	Total (*n* = 1624)	Q1 (*n* = 325)	Q2 (*n* = 325)	Q3 (*n* = 325)	Q4 (*n* = 325)	Q5 (*n* = 324)	
Ethnicity-Han Chinese, n (%)	1013 (62.4)	201 (61.8)	197 (60.6)	203 (62.5)	198 (60.9)	214 (66.0)	0.622
Maternal age, years	31.7 ± 4.0	31.2 ± 4.0	31.5 ± 3.8	31.7 ± 3.9	31.9 ± 4.2	32.1 ± 3.8	0.04
Pre-pregnancy BMI, kg/m^2^	22.4 ± 3.3	21.9 ± 3.0	22.1 ± 3.3	22.4 ± 3.3	22.4 ± 3.5	23.0 ± 3.5	0.004
Assisted reproduction, n (%)	69 (4.2)	6 (1.8)	9 (2.8)	13 (4.0)	18 (5.5)	23 (7.1)	0.007
Abortion history, n (%)	444 (27.3)	63 (19.4)	83 (25.5)	91 (28.0)	98 (30.2)	109 (33.6)	0.001
Gravidity, n (%)							0.039
1	786 (48.4)	184 (56.6)	170 (52.3)	154 (47.4)	145 (44.6)	133 (41.0)	
2	464 (28.6)	84 (25.8)	89 (27.4)	93 (28.6)	97 (29.8)	101 (31.2)	
3	247 (15.2)	39 (12.0)	44 (13.5)	51 (15.7)	54 (16.6)	59 (18.2)	
≥ 4	127 (7.8)	18 (5.5)	22 (6.8)	27 (8.3)	29 (8.9)	31 (9.6)	
Parity, n (%)							0.018
0	1041 (64.1)	219 (67.4)	217 (66.8)	201 (61.8)	201 (61.8)	203 (62.7)	
1	326 (20.1)	70 (21.5)	64 (19.7)	74 (22.8)	66 (20.3)	52 (16.0)	
≥ 2	257 (15.8)	36 (11.1)	44 (13.5)	50 (15.4)	58 (17.8)	69 (21.3)	
Gestational week at examination, weeks	11.1 ± 2.4	10.7 ± 2.5	11.0 ± 2.5	11.4 ± 2.2	11.3 ± 2.2	10.9 ± 2.5	0.002
SBP, mmHg	114.8 ± 9.7	113.6 ± 9.6	114.4 ± 10.6	114.2 ± 9.6	116.0 ± 8.7	116.1 ± 9.8	0.004
DBP, mmHg	69.3 ± 8.8	68.1 ± 7.2	69.0 ± 8.8	69.4 ± 9.4	68.8 ± 8.8	71.0 ± 9.5	0.013
FPG, mg/dL	86.8 ± 8.4	81.9 ± 7.0	85.1 ± 6.2	86.5 ± 7.3	86.8 ± 7.1	93.8 ± 9.2	< 0.001
TG, mg/dL	156.3 (107.2, 226.8)	68.6 (54.6, 83.9)	116.7 (106.8, 126.6)	156.6 (143.3, 168.9)	211.2 (195.7, 228.6)	314.5 (277.4, 369.8)	< 0.001
TC, mg/dL	229.6 ± 53.1	183.3 ± 47.6	228.6 ± 38.3	239.2 ± 44.1	242.0 ± 41.6	254.8 ± 60.8	< 0.001
LDL-C, mg/dL	96.3 ± 40.8	97.6 ± 31.9	103.8 ± 32.3	104.7 ± 41.2	95.2 ± 40.1	80.2 ± 50.7	< 0.001
HDL-C, mg/dL	74.3 ± 17.8	65.3 ± 18.1	77.7 ± 17.5	79.1 ± 15.5	76.6 ± 16.9	73.0 ± 17.6	< 0.001
Hemoglobin, g/L	125.3 ± 15.5	122.8 ± 14.5	125.2 ± 15.7	126.4 ± 15.9	124.8 ± 16.0	127.3 ± 15.2	0.001
Uric acid, μmoI/L	271.8 (225.0, 325.0)	236.0 (199.5, 279.7)	274.7 (229.9, 321.7)	277.6 (231.6, 333.4)	276.2 (231.4, 323.3)	301.1 (251.4, 359.7)	< 0.001
Creatinine, μmoI/L	48.0 (42.9, 53.9)	48.0 (43.0, 54.0)	48.5 (43.2, 53.9)	47.1 (42.0, 53.6)	48.0 (42.7, 53.8)	48.7 (43.3, 54.7)	0.177
ALT, U/L	12.0 (10.0, 16.0)	11.0 (9.6, 14.0)	11.2 (10.0, 14.6)	12.8 (10.3, 16.0)	12.7 (10.0, 16.0)	14.0 (11.0, 18.0)	< 0.001
AST, U/L	14.5 (11.0, 19.2)	13.4 (10.3, 16.9)	14.4 (10.8, 18.5)	15.0 (11.5, 19.2)	14.5 (10.9, 19.8)	16.2 (11.8, 22.1)	< 0.001
Delivery gestations, weeks	38.9 ± 1.1	39.0 ± 1.0	39.0 ± 1.1	38.8 ± 1.2	38.9 ± 1.1	38.8 ± 1.1	0.021
Gestational weight gain, kg	15.4 ± 4.7	14.7 ± 4.6	14.8 ± 4.7	16.0 ± 4.7	16.0 ± 4.9	15.3 ± 4.4	< 0.001
Gestational weight gain, n (%)							< 0.001
< recommendation	89 (5.5)	28 (8.6)	19 (5.8)	16 (4.9)	10 (3.1)	16 (4.9)	
Within recommendation	601 (37.0)	125 (38.5)	147 (45.2)	99 (30.5)	115 (35.4)	115 (35.5)	
> recommendation	934 (57.5)	172 (52.9)	159 (48.9)	210 (64.6)	200 (61.5)	193 (59.6)	

Data are presented as mean ± standard deviation, median (interquartile range) or n (%)

*Abbreviations*: *SBP* Systolic blood pressure, *DBP* Diastolic blood pressure, *FPG* Fasting plasma glucose, *TG* Triglycerides, *TC* Total cholesterol, *LDL-C* Low density lipoprotein cholesterol, *HDL-C* High density lipoprotein cholesterol, *ALT* Alanine aminotransferase, *AST* Aspartate aminotransferase

### TyG index and GDM risk

The logistic regression analysis results highlight the association between the TyG index and the GDM risk (Table 2). In Model 1, adjusted for ethnicity, pre-pregnancy BMI, maternal age, assisted reproduction, abortion history, gravidity, parity, gestational age at delivery, and GWG, each 1-unit increase in the TyG index elevated the GDM risk by 2.10-fold. The highest quintile group exhibited the most significant GDM risk compared with the lowest quintile [odds ratios: 3.25; 95% CI: 2.23–4.75]. These associations were more pronounced in Model 2, which was further adjusted for gestational age at examination, SBP, DBP, TC, LDL-C, HDL-C, hemoglobin, UA, urine creatinine, ALT, and AST. Across all models, a progressive increase in GDM risk was observed with TyG index quintiles (linear trend *P* < 0.001).

**Table 2:** Association between TyG index and GDM

Predictor	Number	GDM, n (%)	Crude		Model 1^a^		Model 2^b^	
			OR (95% CI)	*P* value	OR (95% CI)	*P* value	OR (95% CI)	*P* value
TyG index, per unit	1624	447 (27.5)	2.30 (1.89–2.78)	< 0.001	2.10 (1.72–2.56)	< 0.001	2.81 (2.10–3.77)	< 0.001
TyG index, per SD	1624	447 (27.5)	1.67 (1.48–1.87)	< 0.001	1.58 (1.40–1.78)	< 0.001	1.89 (1.58–2.26)	< 0.001
TyG index Quintile 1	325	53 (16.3)	1 (Ref)		1 (Ref)		1 (Ref)	
Quintile 2	325	68 (20.9)	1.36 (0.91–2.02)	0.131	1.30 (0.87–1.94)	0.206	1.44 (0.93–2.23)	0.105
Quintile 3	325	80 (24.6)	1.68 (1.14–2.47)	0.009	1.54 (1.03–2.29)	0.034	1.67 (1.07–2.61)	0.025
Quintile 4	325	108 (33.2)	2.55 (1.76–3.71)	< 0.001	2.32 (1.58–3.40)	< 0.001	2.82 (1.80–4.42)	< 0.001
Quintile 5	324	138 (42.6)	3.81 (2.64–5.50)	< 0.001	3.25 (2.23–4.75)	< 0.001	3.87 (2.34–6.38)	< 0.001
Trend test				< 0.001		< 0.001		< 0.001

^a^Model 1 adjusted for ethnic, pre-pregnancy BMI, maternal age, assisted reproduction, abortion history, gravidity, parity, delivery gestations, and GWG

^b^Model 2 further adjusted for gestational week at the examination, SBP, DBP, TC, LDL-C, HDL-C, Hb, UA, Cr, ALT and AST

**Fig. 1:**
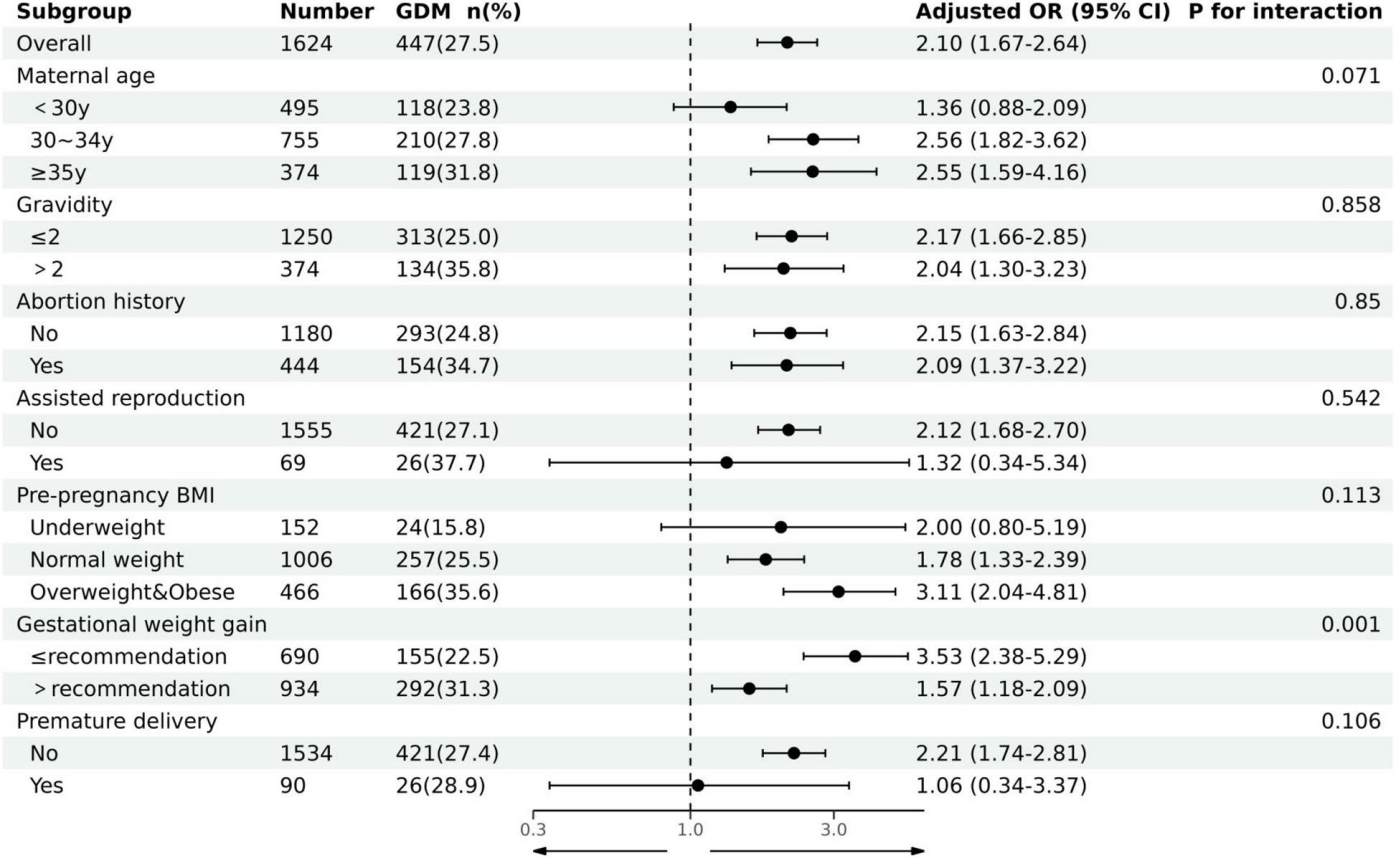
Subgroup analysis of the association between TyG index and GDM risk. Models adjusted for ethnic, pre-pregnancy BMI, maternal age, assisted reproduction, abortion history, gravidity, parity, GWG, delivery gestations, gestational week at the examination, SBP, DBP, TC, LDL-C, HDL-C, Hb, UA, Cr, ALT, and AST

Subgroup analyses based on various factors were conducted to further understand the association between the TyG index and GDM risk. A significant interaction was observed between GWG and the TyG index’s association with GDM risk, particularly when GWG exceeded recommended ranges (*P* = 0.001) (Fig. 1).

### ROC curve analyses of TyG index, maternal age, pre-pregnancy BMI, and FPG in predicting GDM

**Fig. 2:**
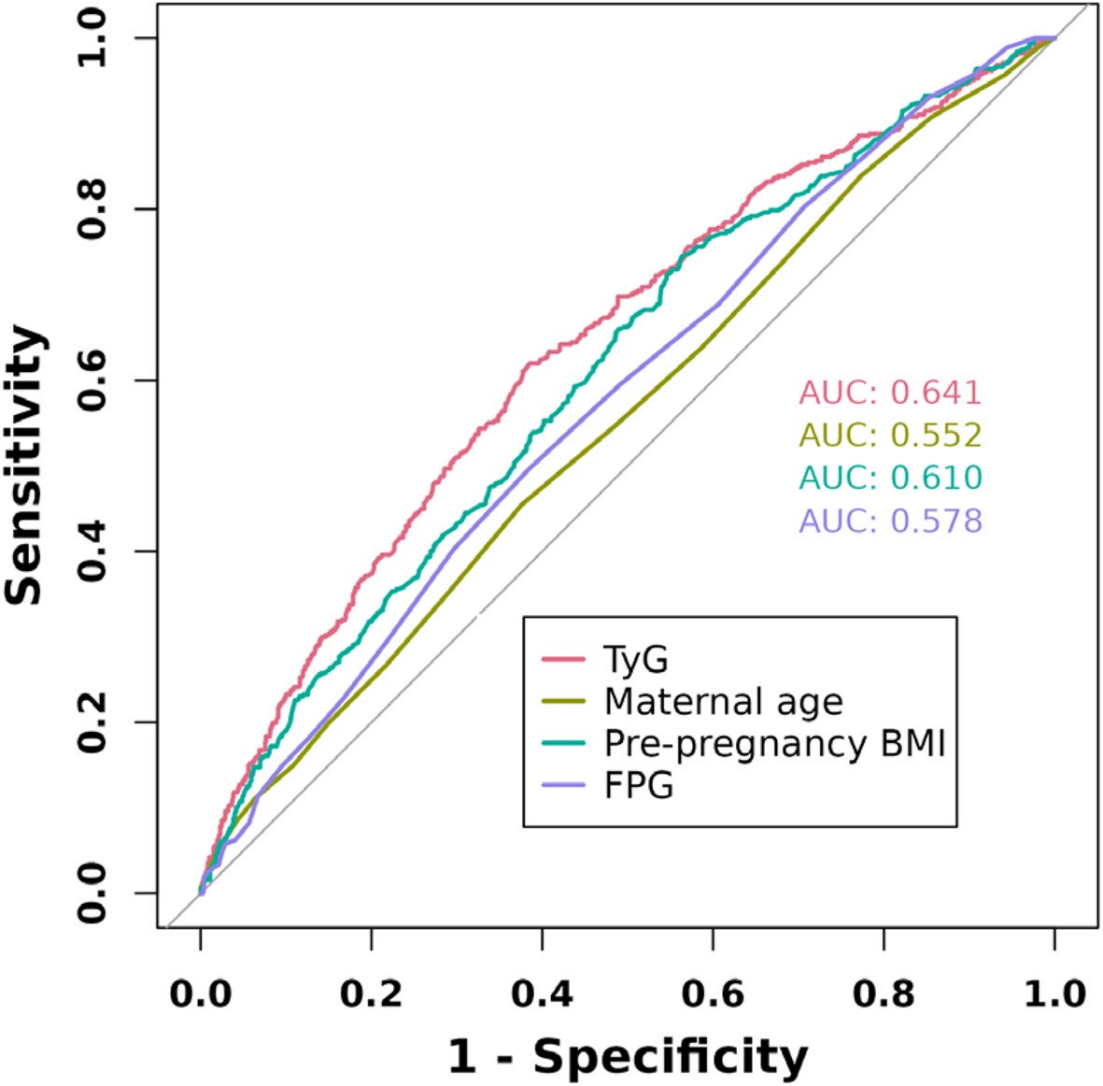
ROC curves of TyG index, maternal age, pre-pregnancy BMI and FPG for predicting the development of GDM

**Fig. 3:**
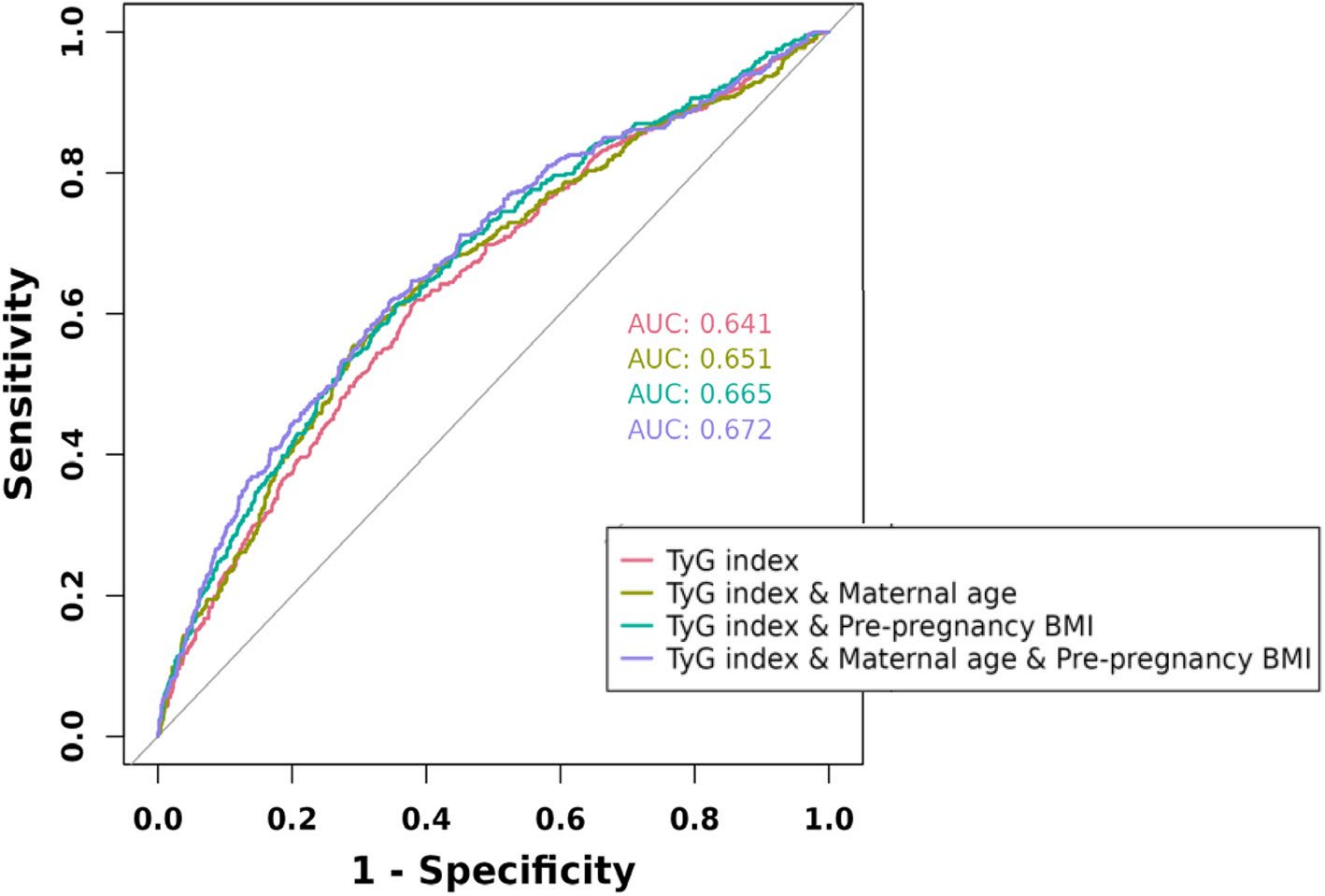
ROC curve of TyG index combined with maternal age and pre-pregnancy BMI for predicting the development of GDM

The independent predictive ability of the TyG index, FPG, maternal age, and pre-pregnancy BMI for GDM was assessed using ROC curves and AUC values. Using the DeLong method, the TyG index was found to be superior to the others (AUC: 0.641, 95% CI: 0.611–0.671), with an optimal cutoff value of 8.890 and both sensitivity and specificity of 0.617 (Fig. 2, Supplementary Table 1). Additionally, ROC curves were generated to compare the predictive ability of the combined prediction of the TyG index with maternal age and pre-pregnancy BMI for GDM. The combined prediction was found to be superior to the predictive ability of the TyG index alone (*P* = 0.00149) (Table 3, Fig. 3).

**Table 3:** Results of ROC analysis of the TyG index used to predict the development of GDM combined with maternal age and pre-pregnancy BMI

Predictor	AUC (95% CI), %	Threshold	Sensitivity	Specificity	PPV	NPV	Accuracy	Precision	Youden’s index	Chi-squared *P* value
TyG index	64.1 (61.1–67.1)	8.890	0.617	0.617	0.380	0.809	0.617	0.380	0.234	< 0.001
TyG index & Maternal age	65.1 (62.1–68.2)	0.300	0.555	0.709	0.420	0.807	0.666	0.420	0.263	< 0.001
TyG index & Pre-pregnancy BMI	66.5 (63.5–69.5)	0.303	0.535	0.719	0.419	0.803	0.668	0.419	0.253	< 0.001
TyG index & Maternal age & Pre pregnancy BMI	67.2 (64.3–70.2)	0.372	0.362	0.867	0.508	0.782	0.728	0.508	0.229	< 0.001

*Abbreviations*: *AUC* Area under curve, *PPV* Positive predictive value, *NPV* Negative predictive value

### TyG index and risk of other PRCs

After adjusting for confounding factors, the multivariate logistic regression analysis indicated a significant 67% increase in the risk of GH and a 58% increase in the risk of preterm rupture of membranes for each 1-unit rise in the TyG index (*P* < 0.05). However, following combined adjustments, only the risk of GH retained significance. The restricted cubic spline plots further demonstrated an escalating risk of GH with an increasing TyG index (Supplementary Fig. 2). No association was found between the TyG index and the incidence of preeclampsia, placental abruption, and fetal distress in any of the models (Supplementary Table 2).

## Discussion

This prospective cohort study aimed to investigate the association between the TyG index during early pregnancy and the risk of PRCs, particularly GDM, among pregnant women in Northwest China. The results revealed a robust and linear correlation between the TyG index and the likelihood of GDM in singleton pregnancies, with an identified optimal cutoff value of 8.890. Additionally, a potential association between the TyG index and the risk of GH was observed. Importantly, combining the TyG index with maternal age and pre-pregnancy BMI showed superior predictive power than the TyG index alone in forecasting the onset of GDM.

GDM is typically diagnosed between the 24th and 28th weeks of pregnancy, providing a limited window for prevention and mitigation of potential adverse effects. Early identification of women at risk for GDM is crucial, as its onset is linked to IR and diminished pancreatic β-cell secretion [7]. However, standard tests for IR, such as the glucose-hyperinsulinism clamp and the homeostatic model assessment of insulin resistance (HOMA-IR), have limitations due to clinical invasiveness, complexity, and the absence of a definitive threshold.

In 2008, the TyG index emerged as an alternative to HOMA-IR, serving as a surrogate marker for detecting IR in healthy individuals [11]. The calculation of the TyG index requires only two routine biochemical parameters: triglycerides and FPG, both of which are measured during routine early pregnancy screenings, eliminating the need for additional testing or complicated experimental procedures. Subsequent research consistently demonstrated that a high TyG index is associated with an increased risk of conditions such as metabolic syndrome [13], cardiovascular disease [22, 23], and type 2 diabetes mellitus (T2DM) [24]. In a comprehensive 15-year study, Wang et al. [25] analyzed a healthy cohort and highlighted that the TyG index and the emergence of T2DM were significantly associated, pinpointing a critical inflection at a value of 8.51. Among 7,708 Koreans aged between 40 and 69 years, Lee et al. [26] found sex-specific thresholds for the TyG index in predicting T2DM (≥ 8.86 for men and ≥ 8.52 for women). Furthermore, Yoon et al. [27] highlighted that the TyG index outperformed HOMA-IR in detecting T2DM in children and adolescents (AUC: 0.839 vs. 0.645).

Research on the association between the TyG index during early pregnancy and the subsequent GDM risk has yielded diverse results. For instance, an Iranian prospective study involving 954 healthy pregnant women, after adjusting for confounding factors like maternal age, family history of diabetes, and early gestational BMI, found that those with a TyG index in the highest tertile during early pregnancy (TyG index ≥ 8.99) faced a 3.91-fold increased GDM risk compared to those in the lowest tertile (TyG index < 8.31) [19]. Similarly, a Chinese prospective cohort study in Beijing, with 352 singleton pregnancies, determined that individuals in the highest TyG index tertile during early gestation (TyG index ≥ 8.3) had a 3.54 times greater GDM risk than those in the lowest tertile (TyG index < 7.9), after accounting for covariates [28].

Conversely, a study involving 164 Latin American pregnant women in early pregnancy stages found no significant correlation between the TyG index and GDM after adjusting for confounders (RR: 1.03, 95% CI: 0.57–1.88). Although no difference in the TyG index was detected between the GDM and control groups during early pregnancy (GDM group: 8.41 ± 0.35, control group: 8.40 ± 0.39; *P* = 0.95), a notable increase in the TyG index was observed in the GDM group compared with that in the control group between 24 and 28 weeks of gestation (GDM group: 9.01 ± 0.30, control group: 8.73 ± 0.34; *P* < 0.001). The study suggested that assessing the TyG index after 12 weeks but before 24 weeks of gestation might be beneficial for the early identification of those at risk for GDM [29].

The present study aligns with the majority of prior research. After comprehensive correction of confounding factors, the results showed that compared with pregnant women in the lowest quintile (TyG index ≤ 8.273), pregnant women in the highest quintile (TyG index ≥ 9.294) in the early pregnancy period had a 3.87 times increased GDM risk. Additionally, the present study highlights the association between excessive GWG and an increased GDM risk. There is growing evidence of the dangers of excessive weight gain during pregnancy, including causing inflammation of the fetal heart and altering fetal cardiac morphology [30]. The TyG index, as a tool to identify pregnant women at elevated risk for GDM during early pregnancy could be a valuable strategy for healthcare practitioners. Early identification of high-risk individuals extends the intervention window, facilitating timely preventive actions for those more susceptible to GDM. Such measures can comprise dietary interventions, appropriate physical activities, and personalized nutritional counseling starting from the early stages of pregnancy.

Determining the optimal cutoff value for the TyG index to predict GDM during early pregnancy varies across studies. Li et al. [15] proposed a cutoff value of 8.55 for the TyG index in early pregnancy, with a specificity of 67.9% and a sensitivity of 53.5%. Kim et al. [17] identified an optimal cutoff value of 8.15 for the TyG index 2 years preceding the first delivery, demonstrating a sensitivity of 47.0% and specificity of 68.2%. In a meta-analysis by Liu et al. [31], the TyG index during the first prenatal visit exhibited an AUC of 0.686 (95% CI: 0.615–0.756), but no specific cutoff value was specified. The present study indicates that the TyG index, with an optimal cutoff value of 8.89, predicts GDM with an AUC of 0.641 (95% CI: 0.611–0.671). The present consensus on the optimal cutoff value for TyG index in predicting GDM ranges between 8.1 and 8.9. Furthermore, the present study suggests that combining TyG index with maternal age and pre-pregnancy BMI enhances the prediction of GDM risk. Wang et al. concluded that excessive pre-pregnancy BMI in mothers is associated with hyperglycemia and hyperlipidemia in the offspring, as well as inflammation, permanently altering organ structure, function, and homeostasis within the organism [32]. Therefore, early prediction of GDM risk in early pregnancy based on TyG index and pre-pregnancy BMI has great clinical relevance. Notably, individual variations, diverse populations, and differences in experimental techniques can introduce variability in the TyG index. Moreover, current research primarily focuses on Asian and Latin American demographics, warranting further validation through extensive, multicenter cohort studies.

The present analysis reveals a correlation between the TyG index and GH incidence, which may be attributed to hyperinsulinemia causing placental ischemia and hypoxia. Reduced nitric oxide synthesis and disruptions in lipid metabolism can impact prostaglandin E2 production, leading to increased peripheral vascular resistance and elevated blood pressure [33]. Another study supports this, indicating that IR significantly increases the risk of GH, aligning with the present study’s conclusions [34]. However, the present study did not reveal a direct association between the TyG index and conditions such as preeclampsia, placental abruption, fetal distress, or premature rupture of membranes.

### Study strengths and limitations

The present study had a prospective design, which enhanced the reliability of findings by observing events over time and minimizing recall bias. Moreover, the comprehensive nature of the study provided thorough insights into the association between the TyG index during early pregnancy and GDM risk, considering various factors. Meticulous data recording methods were used, ensuring accuracy in collecting blood and urine samples during the first trimester and minimizing potential confounding effects on lipid levels. Additionally, conducting blood tests on an empty stomach within 2 h of morning collection enhances the accuracy of lipid level measurements by minimizing the impact of non-fasting conditions and freeze-thaw cycles.

Nonetheless, limitations to the study exist. First, the study did not consider potential confounders such as economic status, dietary habits, physical activity, sleep patterns, and mental health status, which could influence the study outcomes. Second, the TyG index was measured only once in early pregnancy, and fluctuations throughout pregnancy were not tracked, which might have led to overlook of potential variations in TyG index values throughout gestation. Third, while participants with certain health conditions were excluded, complete certainty about the absence of underlying diseases affecting blood glucose, lipid levels, or insulin secretion may be challenging. Lastly, the study focused on Urumqi, a more developed economic region, and generalizing the current findings to less developed regions may require additional studies with larger cohorts, diverse age ranges, and subgroup analysis by several factors to enhance the external validity of findings. Future research should include populations from less developed regions.

### Future research considerations

1. Addition of other biomarkers beyond the TyG index that may predict GDM risk to compare predictive capacity
2. Longer-term follow-up of both mothers and infants after delivery to assess whether early pregnancy TyG index levels have any associationt on postpartum outcomes
3. Analysis of lifestyle intervention measures to reduce the incidence of GDM in pregnant women with elevated TyG index in early pregnancy

## Conclusion

This study highlights the TyG index’s effectiveness in identifying the development of GDM in the latter half of pregnancy, consistent with most research findings. Therefore, the TyG index serves as a valuable early screening tool and can be incorporated into routine obstetric clinical assessments, making it a powerful instrument for clinicians to evaluate the risk of diabetes in pregnant women during obstetric examinations, facilitating early and proactive interventions for high-risk pregnancies. Implementing such measures can reduce the incidence of GDM, leading to improved overall pregnancy outcomes.

### Supplementary Information

**Supplementary Material 1.**

**Supplementary Material 2.**

**Supplementary Material 3.**

**Supplementary Material 4.**

**Supplementary Material 5.**

## Data Availability

The datasets utilized and/or analyzed during the current study are available from the corresponding author upon reasonable request.

## Abbreviations

GDM: Gestational diabetes mellitus
IR: Insulin resistance
TyG: Triglyceride-glucose
BMI: Body mass index
ROC: Receiver operating characteristic
SBP: Systolic blood pressure
DBP: Diastolic blood pressure
FPG: Fasting plasma glucose
TC: Total cholesterol
LDL-C: Low-density lipoprotein cholesterol
HDL-C: High-density lipoprotein cholesterol
UA: Uric acid
ALT: Alanine aminotransferase
AST: Aspartate aminotransferase
PRCs: Pregnancy-related complications
GWG: Gestational weight gain
AUC: Area under the curve
CI: Confidence interval
HOMA-IR: Homeostatic model assessment of insulin resistance
GH: Gestational hypertension
T2DM: Type 2 diabetes mellitus
