# Correction: Triglyceride-glucose index in early pregnancy predicts the risk of gestational diabetes: a prospective cohort study

**DOI:** 10.1186/s12944-024-02110-3

**Published:** 2024-05-04

**Authors:** Yufeng Guo, Junwen Lu, Mailiman Bahani, Guifeng Ding, Lei Wang, Yuxia Zhang, Huanmei Zhang, Chengyao Liu, Lijun Zhou, Xiaolan Liu, Fangshen Li, Xiaoli Wang, Hong Ding

**Affiliations:** 1 Department of Public Health, Xinjiang Medical University, Urumqi, Xinjiang Uygur Autonomous Region 830000 China; 2 Urumqi Maternal and Child Health Hospital, Urumqi, Xinjiang Uygur Autonomous Region 830000 China; 3 Department of Maternal and Child Nutrition, National Institute for Nutrition and Health, Chinese Center for Disease Control and Prevention, Beijing, 100050 China; 4 Maternal and Child Health Care Hospital of Xinjiang Uygur Autonomous Region, Urumqi, Xinjiang Uygur Autonomous Region 830000 China

**Correction: Lipids in Health and Disease 23, 87 (2024)**

10.1186/s12944-024-02076-2

Following publication of the original article [1], the authors requested to add an additional funding source to the funding section. The updated funding section is given below and the changes have been highlighted in bold typeface.

**Funding**.

This research was supported by the Special Project of Scientific and Technological Basic Resources Survey of the Ministry of Science and Technology of China (Grant No. 2019FY101002) **and the 14-th Five-Year Plan Distinctive Program of Public Health and Preventive Medicine in Higher Education Institutions of Xinjiang Uygur Autonomous Region**. The authors would like to express their sincere gratitude to the funding agencies for their financial support.

The original article [1] has been updated.
