# Supplementary figures and images for: Triglyceride-glucose index in early pregnancy predicts the risk of gestational diabetes: a prospective cohort study
Source: Lipids Health Dis. 2024 Mar 25;23:87. doi: 10.1186/s12944-024-02076-2 (PMC10962154; doi:10.1186/s12944-024-02076-2)

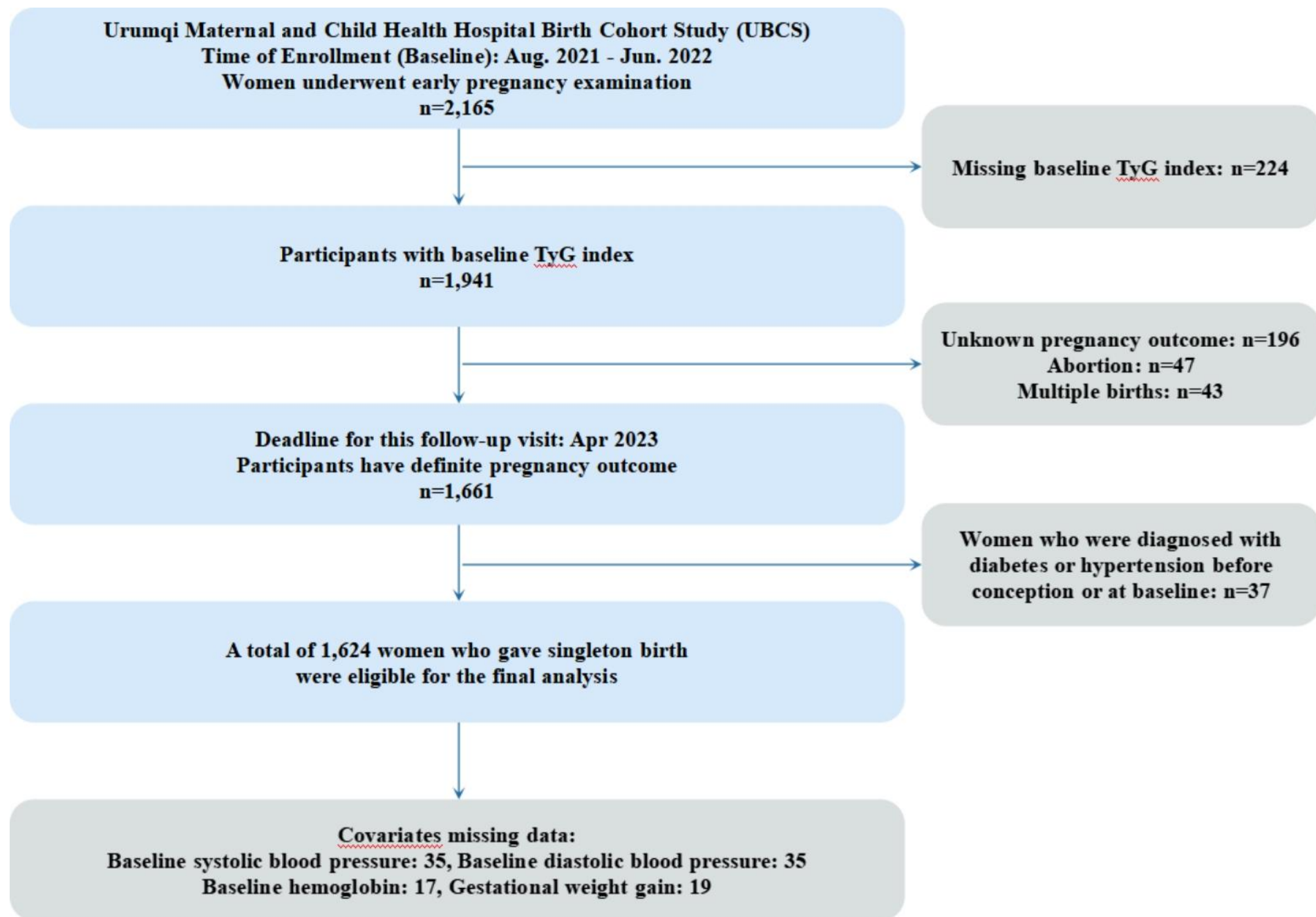

Supplementary Figure 1 Participant flowchart

Supplement: Supplementary file 1 — Supplementary Material 1. [file 12944_2024_2076_MOESM1_ESM.pdf]
