# Supplementary material for: Triglyceride-glucose index in early pregnancy predicts the risk of gestational diabetes: a prospective cohort study
Source: Lipids Health Dis. 2024 Mar 25;23:87. doi: 10.1186/s12944-024-02076-2 (PMC10962154; doi:10.1186/s12944-024-02076-2)
Supplement: Supplementary file 2 — Supplementary Material 2. [file 12944_2024_2076_MOESM2_ESM.pdf]

Supplementary Table 1 Results of receiver operating characteristic analysis of TyG index, Maternal age, Pre-pregnancy BMI and FPG used to predict the development of GDM

Predictor	AUC (95%CI), %	Threshold	Sensitivity	Specificity	PPV	NPV	Accuracy	Precision	Youden's index	Chi-squared *P* value
TyG index	64.1（61.1 - 67.1）	8.890	0.617	0.617	0.380	0.809	0.617	0.380	0.234	＜0.001
Maternal age	55.2（52.1 - 58.3）	39.000	0.085	0.958	0.432	0.734	0.717	0.432	0.043	＜0.001
Pre-pregnancy BMI	61.0（57.9 - 64.0）	20.937	0.745	0.437	0.334	0.818	0.522	0.334	0.182	＜0.001
FPG	57.8（54.7 - 60.8）	79.290	0.931	0.147	0.293	0.848	0.363	0.293	0.078	＜0.001

Abbreviations: AUC, area under curve; PPV, positive predictive value; NPV, negative predictive value.
