# Supplementary material for: Triglyceride-glucose index in early pregnancy predicts the risk of gestational diabetes: a prospective cohort study
Source: Lipids Health Dis. 2024 Mar 25;23:87. doi: 10.1186/s12944-024-02076-2 (PMC10962154; doi:10.1186/s12944-024-02076-2)
Supplement: Supplementary file 3 — Supplementary Material 3. [file 12944_2024_2076_MOESM3_ESM.pdf]

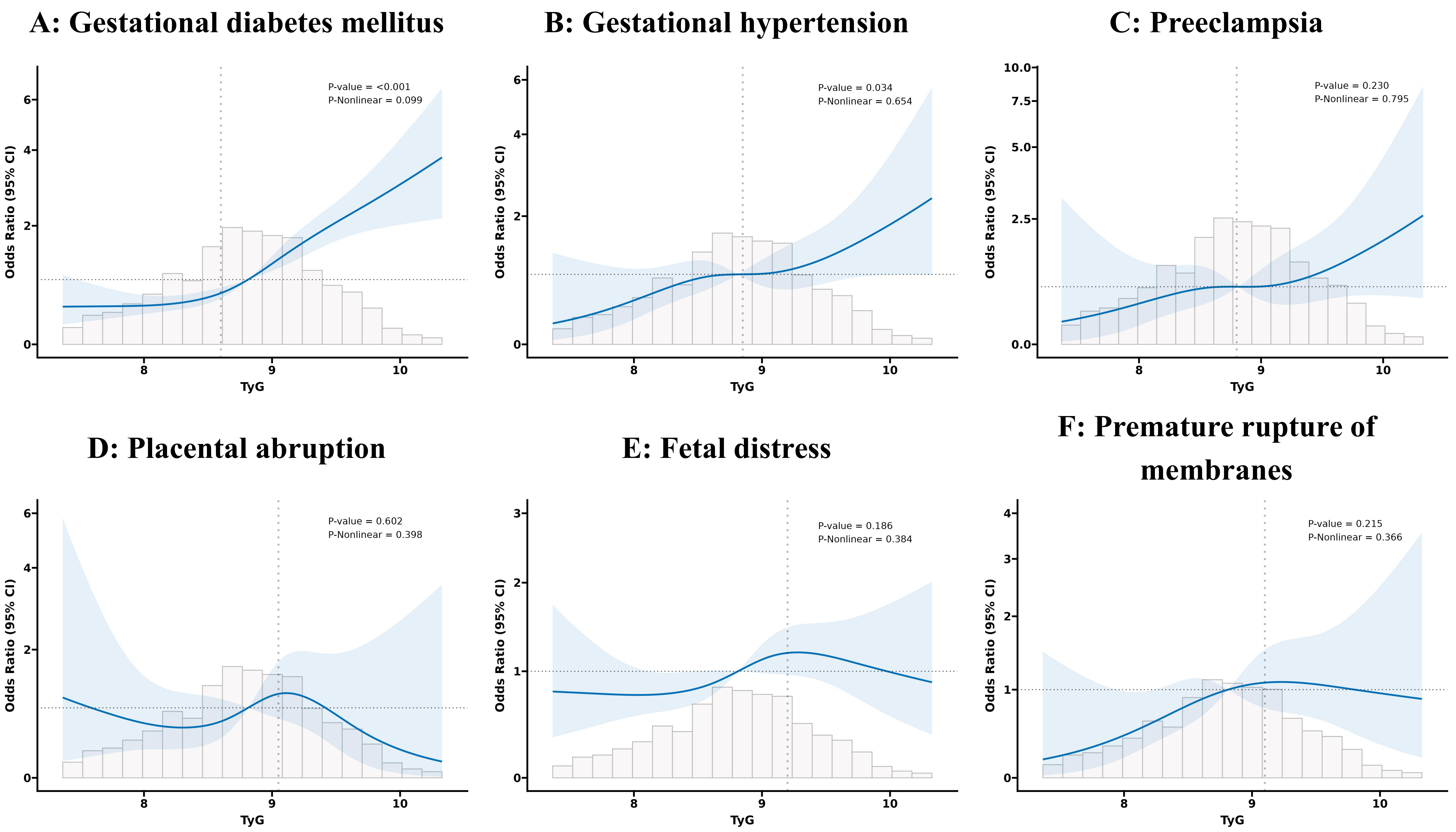

Supplementary Figure 2 The risk of pregnancy-related complications with increasing triglyceride-glucose (TyG) index. The solid line represents adjusted probability and shadow parts 95% confidence intervals. Adjusted for ethnic, pre-pregnancy BMI, maternal age, assisted reproduction, abortion history, gravidity, parity, gestational weight gain, delivery gestations, gestational week at the examination, systolic blood pressure, diastolic blood pressure, total cholesterol, low density lipoprotein cholesterol, high density lipoprotein cholesterol, hemoglobin, uric acid, creatinine, alanine aminotransferase, and aspartate aminotransferase. (A), Gestational diabetes mellitus; (B), Gestational hypertension; (C), Preeclampsia; (D), Placental abruption; (E), Fetal distress; (F), Premature rupture of membranes.
