# Supplementary material for: Triglyceride-glucose index in early pregnancy predicts the risk of gestational diabetes: a prospective cohort study
Source: Lipids Health Dis. 2024 Mar 25;23:87. doi: 10.1186/s12944-024-02076-2 (PMC10962154; doi:10.1186/s12944-024-02076-2)
Supplement: Supplementary file 4 — Supplementary Material 4. [file 12944_2024_2076_MOESM4_ESM.pdf]

Supplementary Table 2 Association of Triglyceride-Glucose (TyG) Index with Other Pregnancy-Related Complications

Predictor	Number	Pregnancy-Related Complications, n (%)	Crude		Model 1 [a]		Model 2 [b]	
			OR (95%CI)	*P value*	*OR (95%CI)*	*P value*	*OR (95%CI)*	*P value*
Gestational hypertension	1624	88（5.4）	1.67 (1.17~2.38)	**0.004**	1.67 (1.16~2.40)	**0.006**	1.87 (1.11~3.15)	**0.019**
Preeclampsia	1624	41（2.5）	1.63 (0.98~2.71)	0.058	1.54 (0.92~2.59)	0.100	1.84 (0.87~3.90)	0.114
Placental abruption	1624	34（2.1）	0.99 (0.57~1.72)	0.970	1.02 (0.58~1.82)	0.941	1.09 (0.49~2.43)	0.826
Fetal distress	1624	209（12.9）	1.23 (0.97~1.56)	0.091	1.19 (0.93~1.51)	0.170	1.36 (0.95~1.94)	0.096
Premature rupture of membranes	1624	60（3.7）	1.49(0.98~2.27)	0.064	1.58 (1.02~2.44)	**0.039**	1.56 (0.83~2.92)	0.169

Notes: [a]Model 1 adjusted for ethnic, pre-pregnancy BMI, maternal age, assisted reproduction, abortion history, gravidity, parity, delivery gestations, and gestational weight gain; [b]Model 2 further adjusted for gestational week at the examination, systolic blood pressure, diastolic blood pressure, total cholesterol, low density lipoprotein cholesterol, high density lipoprotein cholesterol, hemoglobin, uric acid, creatinine, alanine aminotransferase, and aspartate aminotransferase. The bolded P value indicates statistical significance.
