# Supplementary material for: Triglyceride-glucose index in early pregnancy predicts the risk of gestational diabetes: a prospective cohort study
Source: Lipids Health Dis. 2024 Mar 25;23:87. doi: 10.1186/s12944-024-02076-2 (PMC10962154; doi:10.1186/s12944-024-02076-2)
Supplement: Supplementary file 5 — Supplementary Material 5. [file 12944_2024_2076_MOESM5_ESM.pdf]

Supplementary Table 3 ROC Analysis of the Triglyceride-Glucose (TyG) Index for Detecting Pregnancy-Related Complications

Predictor	AUC (*95%CI*), %	Threshold	Sensitivity	Specificity	PPV	NPV	Accuracy	Precision	Youden's index	Chi-squared P value
Gestational diabetes mellitus	64.10(61.10~67.10)	8.890	0.617	0.617	0.380	0.809	0.617	0.380	0.234	＜0.001
Gestational hypertension	57.70(51.80~63.60)	8.605	0.796	0.359	0.066	0.968	0.382	0.066	0.154	0.003
Preeclampsia	57.30(48.50~66.10)	8.574	0.805	0.339	0.031	0.985	0.350	0.031	0.143	0.055
Placental abruption	51.40(41.60~61.10)	9.186	0.382	0.745	0.031	0.983	0.737	0.031	0.127	0.094
Fetal distress	54.40(50.30~58.60)	9.188	0.349	0.758	0.176	0.888	0.706	0.176	0.108	＜0.001
Premature rupture of membranes	57.30(50.40~64.10)	8.735	0.717	0.454	0.048	0.977	0.464	0.048	0.171	0.009

Abbreviations: AUC, area under curve; PPV, positive predictive value; NPV, negative predictive value.
